# Supplementary material for: Low-energy band structure and even-odd layer number effect in AB-stacked multilayer graphene
Source: Sci Rep. 2018 Aug 29;8:13018. doi: 10.1038/s41598-018-31291-y (PMC6115377; doi:10.1038/s41598-018-31291-y)
Supplement: Supplementary file 1 — Supplementary Information [file 41598_2018_31291_MOESM1_ESM.pdf]

Supplementary Information

Low-energy band structure and even-odd layer number effect in AB-stacked multilayer graphene

Ryuta Yagi[1], Taiki Hirahara[1], Ryoya Ebisuoka[1], Tomoaki Nakasuga[1], Shingo Tajima[1], Kenji Watanabe[2], and Takashi Taniguchi[2]

[1] Graduate School of Advanced Sciences of Matter, Hiroshima University, Kagamiyama Higashihiroshima, Hiroshima 739-8530, Japan.
[2] National Institute of Material Science (NIMS) , Namiki, Tsukuba 305-0044, Japan.

Method of sample fabrication

Each sample was graphene encapsulated with high quality *h*-BN flakes, or graphene transferred onto a *h*-BN flake. The graphene was prepared by mechanically exfoliating high-quality Kish graphite onto a SiO_2 substrate by using adhesive tape [1]. The SiO_2 substrate was annealed in atmosphere at approximately 800° C in advance to reduce contamination on its surface. The *h*-BN flakes were also prepared by mechanical exfoliation. The encapsulation technique and transfer technique are described in Refs. [2, 3]. Electric contacts were formed by the lift off technique using electron beam lithography and vacuum evaporation of Cr and Au. In the case of the encapsulated samples, before forming the electrical leads, the contact region was ion-etched using a low-pressure mixture of CF_4 and O_2 gas to remove the top layer of *h*-BN and expose the graphene underneath [2]. In the final fabrication process, the unencapsulated samples were annealed in a mixture of H_2 (about 6 percent) and Ar (94 percent) for approximately six hours at approximately 330 °C, to reduce contamination on the graphene surface. The typical two-terminal resistance was less than approximately 10 kOhm.

Layer number and stacking

The number of layers was determined by topography obtained using an atomic force microscope (AFM), color intensity analysis of an optical micrograph of graphene [4], and Raman spectroscopy [5]. Figure S1 **a** shows an optical micrograph of a graphene flake that has domains with various numbers of layers. Figure S1 **b** shows the AFM topography of the same sample. A height scan taken along a line is shown in panel **c**. The step height was approximately integer multiples of the interlayer distance of graphite, ~ 0.34 nm. These measurements were reproducible, and one could calibrate the relationship between the layer numbers and color intensity of the optical micrograph for the graphene flakes.

We further verified the number of layers and their stacking with Raman spectroscopy. The line shape of the G' mode (G' peak) appearing at approximately 2700 cm^{-1} in the spectra is strongly dependent on the number of layers [5-10]. The Raman spectra for many samples were measured using an excitation laser with a wavelength of 532 nm and with an 100 × objective lens (Raman Plus, Nanophoton Co.). Data were classified according to the shapes of the G' peaks. Figure 1 **d** in the main text shows the results for AB stacked graphene having three to seven layers. In the figure, the spectra were normalized and offset vertically and slightly horizontally to better compare the line shapes. Moreover, the shapes of the Raman G' peaks coincide with different samples having the same number of layers in Fig. S2. In Figs. 1 **d** and S2, the vertical offset from one graph to the next is due to the background signal varying slightly depending on the experimental setup, whereas the slight variations in the center of the spectra could have resulted from internal strain [12, 13]. These data are consistent with those of previous studies on AB stacked graphene with layer numbers from one to five [5, 8]. Although we could not find reports for six- and seven-layer graphene, the Raman G' band peaks have been reported to show systematic variation up to layer 10 and bulk graphite [5], which convinced us that G' peaks from the samples with 6 and 7 layers were due to AB-stacked graphene. Moreover the shapes of the spectra for each layer in Figure S2 were most frequently observed in graphene flakes prepared from natural graphite as well as Kish graphite, which show a dominant ABA stacking. This fact would exclude the possibility that the line shapes in Fig. 1 d and S2 are for ABC-stacked graphene and not for samples with AB-stackings.

Figure S3 shows the relationship between the number of layers and the magnitude of the G peak appearing at 1570 cm^{-1}. The magnitude of the peak showed systematic variation with the number of layers as determined from other methods [6, 14]. The normalized G peak intensity increases approximately linearly, as was reported in Refs. [6, 14].

Domains with different stackings

The shape of the Raman G' peak reveals the kind of stacking [6, 10]. However, the stacking may vary within a graphene flake. Here, different stackings within a graphene flake can be detected by conducting a Raman mapping experiment [6, 7]. Lui *et al.* determined the stacking order of graphene with a few layers from the spectral widths of the Raman G' band peaks. In particular, they exploited the fact that the Raman G' band spectra of ABC-stacked graphene show Raman G' band spectra that are different in shape from those for AB-stacked graphene [6, 10]. In our experiments we used an easier method to map out the different layers and stackings by using the signal intensity at a fixed Raman shift value in the vicinity of the G peak. This method is based on the fact that the G peak is sensitive to not only the layer number but also the stacking order [6, 10], because it results from the electronic band structure of graphene. Figure S4 shows an example. Panel **a** shows an optical micrograph of multilayer graphene that has domains with different layer numbers. Panel **b** shows the mapping plot of the Raman spectral intensity for a bin between 1573 and 1575 cm^{-1}. Let us focus on the left-most domain in panel **a**, which is four-layer. In the optical micrograph, it appears that the domain to the left of the dashed line has four layers. However in the mapping plot (panel **b**), the domain appears to consist of different regimes *X*, *Y* and *Z*. The intensity of the G band spectra for *Y* is apparently larger than that for *X* and *Z*, as shown in Panel **c** and the Raman peak shifts to smaller values. Moreover the shape of the G' band spectra was clearly different from those of *X* and *Y*, as can be seen in Panel **d**. Using line shapes obtained from preceding studies, [10, 6] regime *X* could be identified to be ABAB-stacking, while regime *Y* was identified to be ABCA-stacking. As for regime *Z*, the shape of the G' band spectra was close to that of regime *X*, albeit with a little smaller intensity; it might be ABAC stacking, which has not been reported elsewhere.

Method of calculation

The dispersion relation of the electronic band in zero magnetic fields was calculated using the effective mass approximation taking into account all the parameters in the Slonczewski-Weiss-McClure (SWMcC) model [17]. The definitions of these parameters are shown in Fig. S5. We expanded the wave function of graphene by using plane waves and calculated the eigenvalues numerically. The areas of equal-energy contours for the dispersion relation were calculated and converted into carrier densities under the assumption that the band has a degeneracy of 4 arising from spins and valleys. The SWMcC parameters used in the calculation were $\gamma_0 =$ 3.0 eV, $\gamma_1 = 0.45$ eV, $\gamma_2 = -0.023$ eV, $\gamma_3 = 0.3$ eV, $\gamma_4 = 0.04$ eV, $\gamma_5 =$ 0.04 eV, and $\Delta_\mathrm{p} = 0.032$ eV. The calculation took into account the variation of the electric potential energy resulting from carriers induced in graphene by the external gate voltage and causing the carrier density to vary with it. Here, we simply assumed that the induced carrier density decayed exponentially with a characteristic decay length, as expected in Thomas-Fermi model [18]. Accordingly, carriers in each layer in N-layer graphene can be expressed using the decay length λ as

$$n_\mathrm{j} = n_0\, \mathrm{e}^{-\frac{aj}{\lambda}} \ . \tag{S1}$$

(see Fig. S6). Here, $j = 1,2,3 \ldots N$ is a natural number which was counted from the substrate side of multilayer graphene, and a is the inter-layer distance of graphene. The variable n_0 is readily described as

$$n_0 = \frac{n_{tot}}{\sum_{j=1}^{N} \mathrm{e}^{-\frac{aj}{\lambda}}} \ . \tag{S2}$$

If we ignore parasitic capacitance, we may safely assume that the electric field outside the graphene beyond layer $j = N$ vanishes. By solving the Maxwell equations, the electric field component between the jth and the $(j+1)th$ layer and perpendicular to the graphene plane is given by

$$E_{j,j+1} = -\frac{n_{tot} - \sum_{j'=j+1}^{N} n_{j'}}{\epsilon\epsilon_0}, \tag{S3}$$

where ϵ_0 is the dielectric constant of vacuum and ϵ is the relative dielectric constant of graphene, which we took as $\epsilon = 2$. The difference in the electric potential energy between the jth layer and the $(j+1)$th layer, *i.e.*, $-e(\phi_{j+1} - \phi_j)$ is then

$$-e(\phi_{j+1} - \phi_j) = ea\left(\frac{n_{tot} - \sum_{j'=j+1}^{N} n_{j'}}{\epsilon\epsilon_0}\right) . \qquad \text{(S4)}$$

We took layer $j = 1$ to be the reference point for zero energy, and calculated the potential energy of each layer. These potential energy values were added to the diagonal elements in the Hamiltonian.

Carrier screening has significant effects in experiments on samples with a single gate electrode. In our experiment, the total carrier density was tuned up to $|n_{tot}| \sim 3 \times 10^{12}$ cm^{-2} for one- to four-layer graphene and $|n_{tot}| \sim 5 \times 10^{12}$ cm^{-2} for six- and seven-layer graphene. For one-, two-, three- and four-layer graphene, we could obtain approximate agreement between theory and experiment without considering the carrier distribution. However, for a number of layers $N \geq 5$, the dependence of n_{osc} on n_{tot} could not be explained unless the screening effect was considered. Figure 3 in the main text shows that λ was 0.33, 0.33, 0.33, 0.43, and 0.35 nm for graphene with $N = 3 \sim 7$, respectively. The experimental result cannot be explained without the screening effect. Figure S7 shows the result of a calculation not considering carrier screening. Here, the SWMcC parameters are the same as those of graphite, $\gamma_0 = 3.16$ eV, $\gamma_1 = 0.39$ eV, $\gamma_2 = -0.02$ eV, $\gamma_3 = 0.31$ eV, $\gamma_4 = 0.04$ eV, $\gamma_5 = 0.038$ eV, and $\Delta_p = 0.037$ eV. Tuning of the parameters did not significantly alleviate the discrepancy between theory and experiment.

On higher order harmonics in FFT

In Fig. 3 in the main text, conspicuous peak structures other than those for the fundamental frequency were observed due to higher order harmonics, whose frequency could be explained by the following particularly simple arithmetic for the basic components. Figures S8, S9 and S10 show examples of FFT spectra for two- to seven-layer graphene for various values of carrier density. For example, in the case of four-layer graphene (Fig. S9), fb_1 and fb_2 correspond to the fundamental

frequencies of a light-mass bilayer band and a heavy-mass bilayer band. Moreover, frequencies of the small peaks were found to be fb_1-fb_2, $2fb_2$ and $fa = fb_1+fb_2$. Note that there are no physical orbits that correspond to these peaks. These spectra are higher order harmonics, which possibly stem from mixing of the fundamental frequencies possibly due to non-linearity.

References

[1] Novoselov, K. S. *et al.*, Two-dimensional gas of massless Dirac Fermions in graphene, Nature **438**, 197-200 (2005).

[2] Wang, L. *et al.,* One-dimensional electrical contact to a two-dimensional material, Science **342**, 614-617 (2013).

[3] Taychatanapat, T., Watanabe, K., Taniguchi, T. & Jarillo-Herrero, P., Quantum Hall effect and Landau-level crossing of Dirac Fermions in trilayer graphene, Nature Phys. **7**, 621-625 (2011).

[4] Blake, P. *et al.*, Making graphene visible, Appl. Phys. Lett. **91**, 063124 (2007).

[5] Ferrari, A. C. *et al.*, Raman spectrum of graphene and graphene layers, Phys. Rev. Lett. **97**, 187401 (2006).

[6] Lui, C. H. *et al.,* Imaging stacking order in few-layer graphene, Nano Lett. **11**, 164-169 (2011).

[7] Graf, D. *et al.*, Spatially resolved Raman spectroscopy of single- and few-layer graphene, Nano Lett. **7**, 238-242 (2007).

[8] Mak, K. F., Shan, J. & Heinz, T. F., Electronic structure of few-layer graphene, Phys. Rev. Lett. **104**, 176404 (2010).

[9] Mak, K. F., Sfeir, M. Y., Misewich, J. A. & Heinz, T. F., The evolution of electronic structure in few-layer graphene revealed by optical spectroscopy, Proc. Nat. Acad. Sci. **107**, 14999-15004 (2010).

[10] Cong, C. X. *et al.*, Raman characterization of ABA- and ABC-stacked trilayer graphene, **107**, 5 (8760-8768).

[11] Hao, Y. F. *et al.*, Probing layer number and stacking order of few-layer graphene by Raman spectroscopy, Small **6**, 195-200 (2010).

[12] Mohiuddin, T. M. G. *et al.*, Uniaxial strain in graphene by Raman spectroscopy, Phys. Rev. B **79**, 205433 (2009).

[13] Zabel, J. *et al.*, Raman spectroscopy of graphene and bilayer under biaxial strain, Nano Lett. **12,** 617-621 (2012).

[14] Gupta, A., Chen, G., Joshi, P., Tadigadapa, S. & Eklund, P. C., Raman scattering from high-frequency phonons in supported N-graphene layer films, Nano Lett. **6**, 2667-2673 (2006).

[15] Nguyen, T. A., Lee, J. U., Yoon, D. & Cheong, H., Excitation energy dependent Raman signatures of ABA- and ABC-stacked few-layer graphene, Sci. Rep. **4**, 4630 (2014).

[16] Koshino, M. & Ando, T., Electronic structures and optical absorption of multilayer graphenes, Solid State Commun. **149**, 1123-1127 (2009).
[17] Divincenzo, D. P. & Mele, E. J., Self-consistent effective-mass theory for intralayer screening in graphite-intercalation compounds, Phys. Rev. B **29**, 1685-1694 (1984).

Figure Legends

Fig. S1 Determination of the number of layers by AFM. |

a Optical micrograph of a graphene flake that has domains with various numbers of layers. **b** Result of AFM topography. **c** Scan along the blue line indicated in panel **b**. Step height is approximately an integer multiples of the inter layer distance of graphite, approximately 0.34 nm.

Fig. S2 Reproducibility of Raman G' peak data. |

Raman G' band spectra for graphene with different numbers of layers. For each layer number, spectra of different samples were plotted to confirm reproducibility in line shapes from sample to sample. The magnitude of the spectra was offset and normalized. The Raman shift was also slightly offset to compare the line shapes of different samples.

Fig. S3 Relation between layer number and Raman G-band peak intensity. |

Raman G-band peak intensity is plotted as a function of calibrated layer number, which was determined from the G' band line shape and color intensity analysis.

Fig. S4 Example of a Raman mapping experiment. |

a Optical micrograph of a graphene sample composed of regions with different numbers of layers. The region left of the dashed line is the four-layer. **b** Mapping of the Raman spectra intensity for a bin between 1573 and 1575 cm^{-1}. **c** Raman G-band spectra for regimes X (green), Y (red), and Z (blue). **d** Raman G'-band spectra for the same regimes.

Fig. S5 Definition of the SWMcC parameters. |

Fig. S6 Screening in a graphene sample with a single gate electrode. |

Model of screened gate electric fields and induced carriers in multilayer graphene. λ is the screening length. n_j ($j = 1$ to N) is the carrier density in the j-th layer of N-layer graphene.

Fig. S7 Calculation without screening of gate-induced carriers. |

The experimentally obtained carrier density for each band is compared with a calculation not considering the screening effect. The dispersion relation was

calculated using the SWMcC parameters of graphite. Agreement between theory and experiment was poor for $N \geq 5$. Fine tuning of the SWMcC parameters did not qualitatively change the result.

Fig. S8 FFT spectra of magnetoresistance. |
Results for monolayer and trilayer graphene. The frequency of the FFT spectra was multiplied by a factor $4\,(e/h)$ so as to indicate the carrier density of the Fermi surface of the bands relevant to the Shubnikov-de Haas effect. fa is a signal for the total carrier density, fm is for monolayer bands, fb_1, fb_2 and fb_3 are for bilayer bands.

Fig. S9 FFT spectra of magnetoresistance. |
Similar to the plot shown in Fig. S8 but for four- and five-layer graphene.

Fig. S10 FFT spectra of magnetoresistance. |
Similar to the plot shown in Fig. S8 but for six- and seven- layer graphene.

Fig. S1

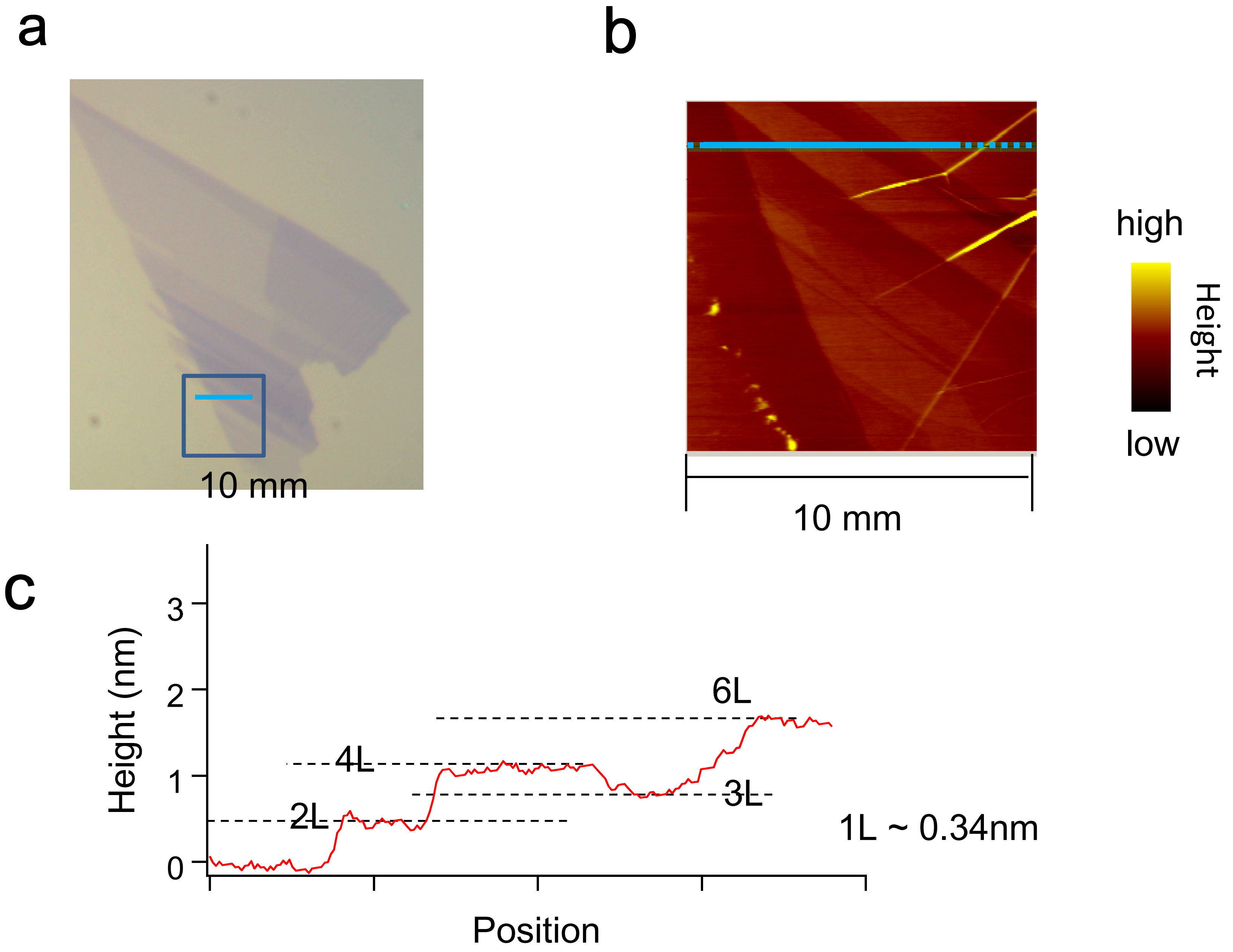

a
10 mm
b
10 mm
high
Height
low
c
Height (nm)
3
2
1
0
Position
6L
4L
3L
2L
1L ~ 0.34nm

Fig. S2

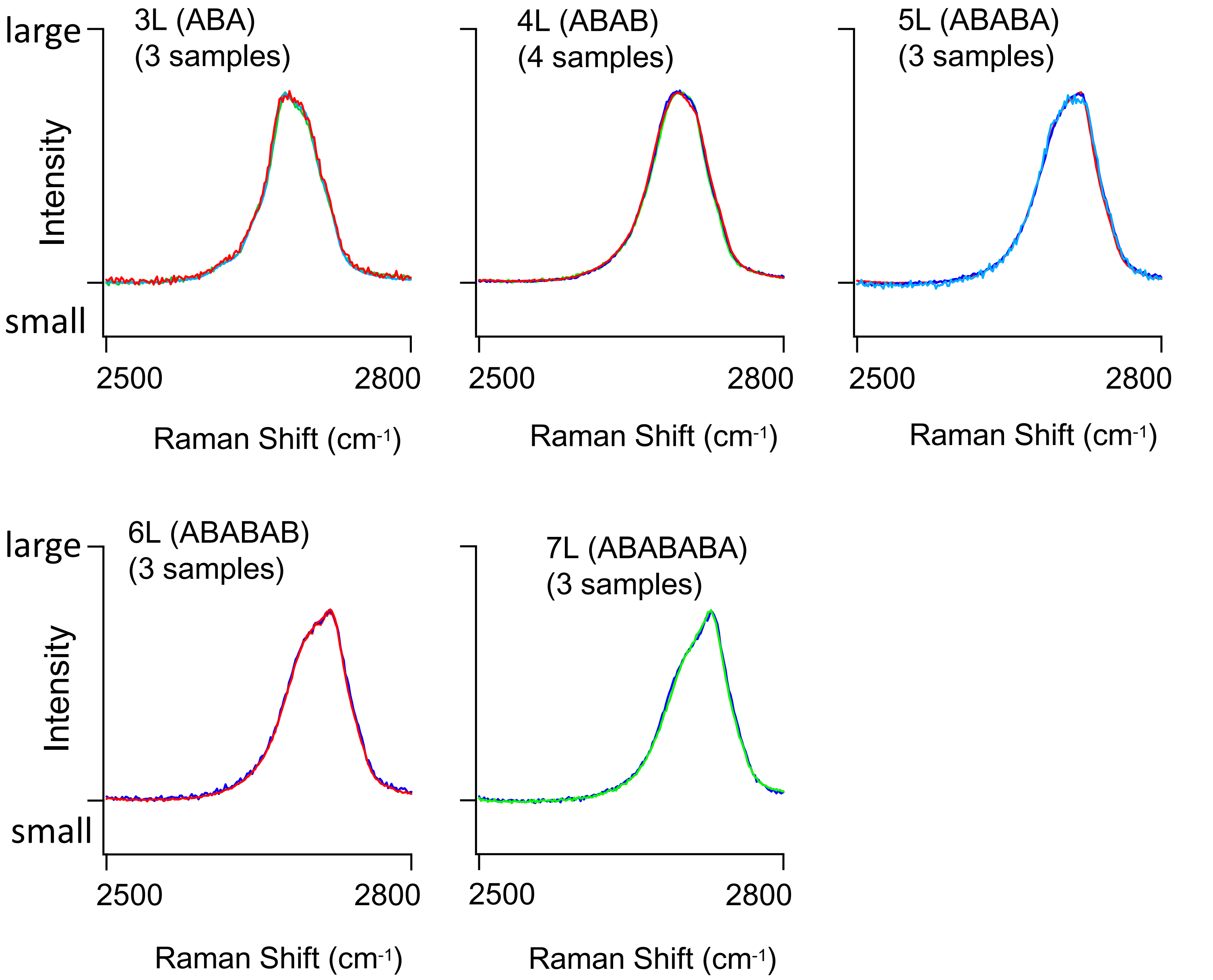

3L (ABA)
(3 samples)
4L (ABAB)
(4 samples)
5L (ABABA)
(3 samples)
6L (ABABAB)
(3 samples)
7L (ABABABA)
(3 samples)
large
small
Intensity
2500
2800
Raman Shift (cm-1)

Fig. S3

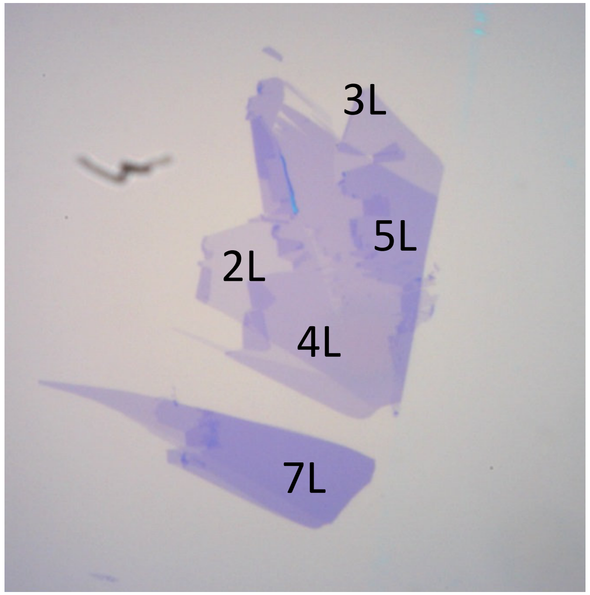
3L
5L
2L
4L
7L

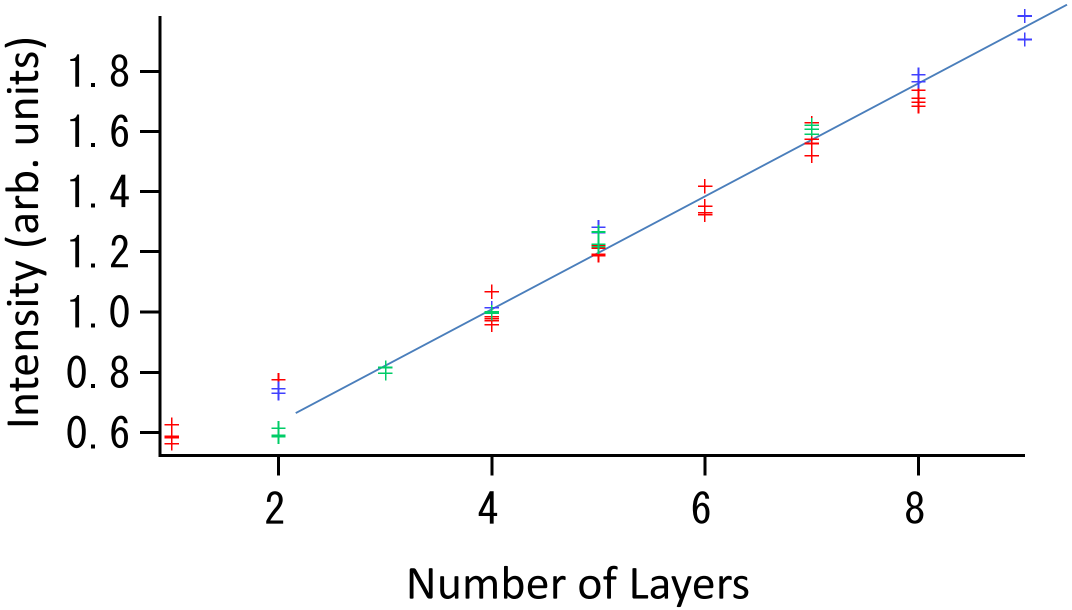
Intensity (arb. units)
1.8
1.6
1.4
1.2
1.0
0.8
0.6
2
4
6
8
Number of Layers

Fig. S4

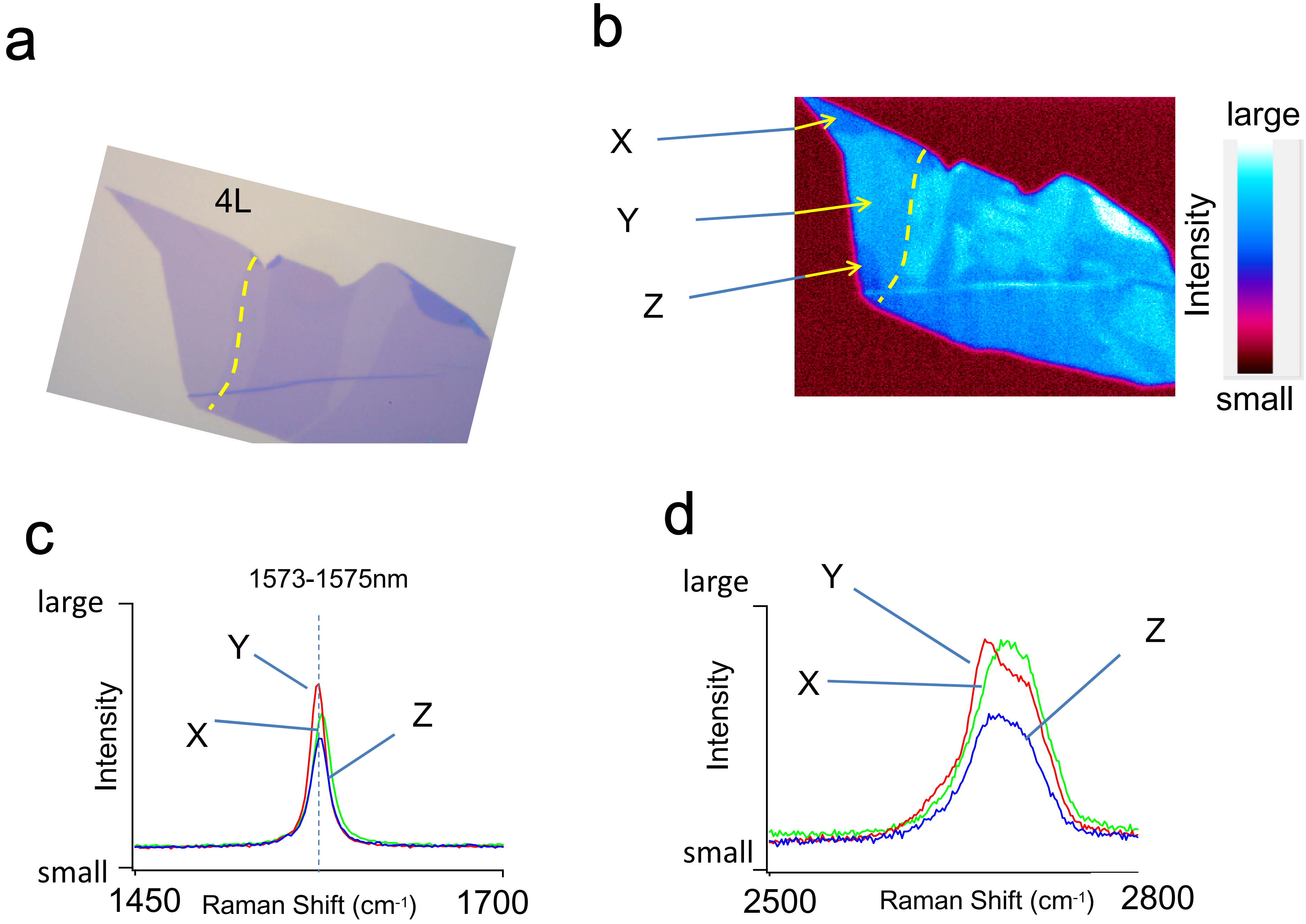
a
4L
b
X
Y
Z
large
Intensity
small
c
1573-1575nm
large
Intensity
small
Y
X
Z
1450
Raman Shift (cm-1)
1700
d
large
Intensity
small
Y
X
Z
2500
Raman Shift (cm-1)
2800

Fig. S5

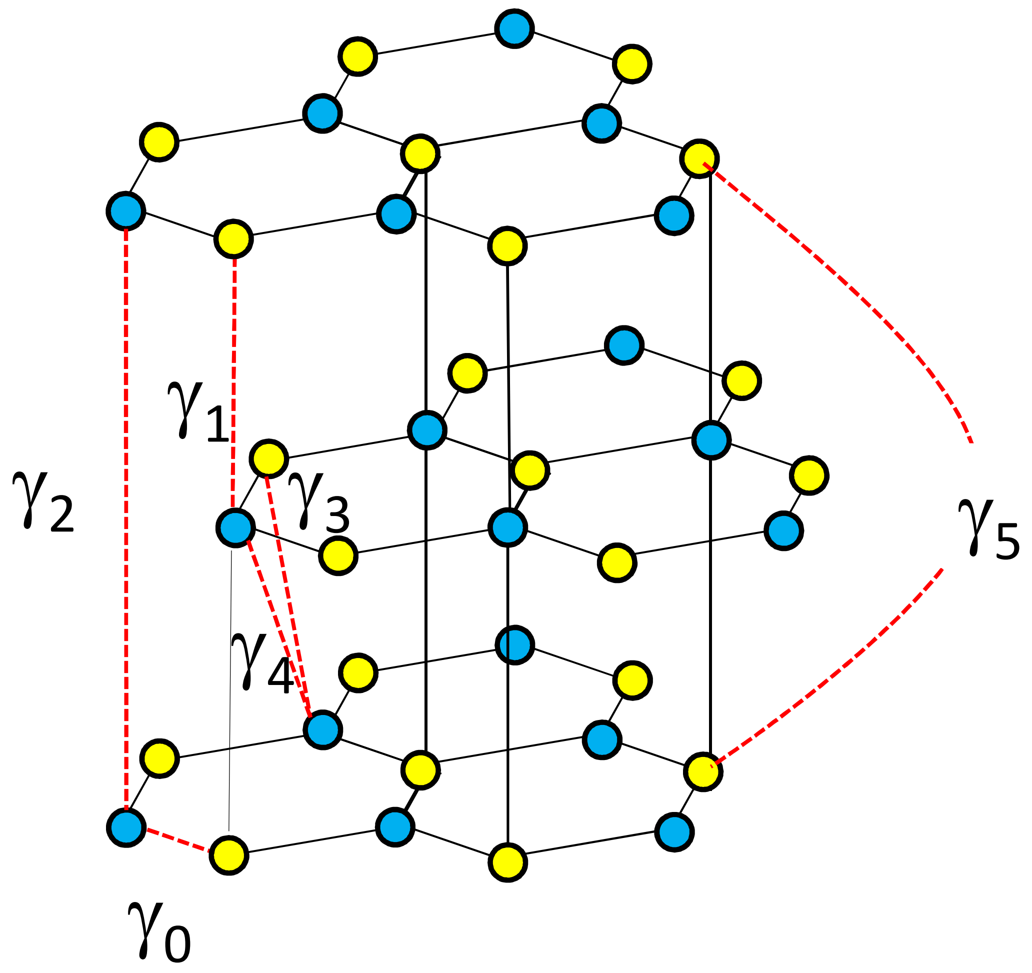

Fig. S6

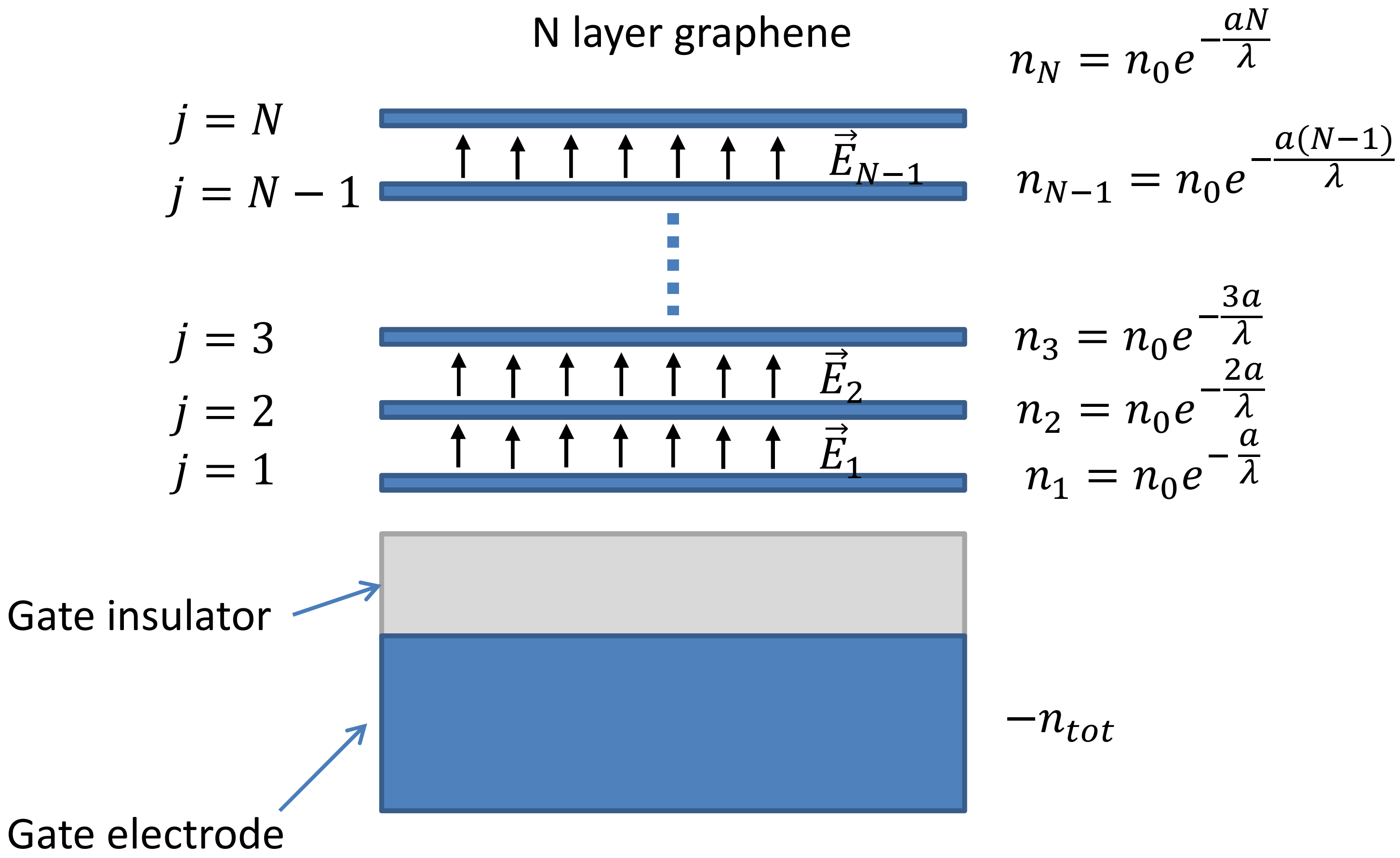
N layer graphene
$j = N$
$j = N - 1$
$j = 3$
$j = 2$
$j = 1$
$\vec{E}_{N-1}$
$\vec{E}_2$
$\vec{E}_1$
$n_N = n_0 e^{-\frac{aN}{\lambda}}$
$n_{N-1} = n_0 e^{-\frac{a(N-1)}{\lambda}}$
$n_3 = n_0 e^{-\frac{3a}{\lambda}}$
$n_2 = n_0 e^{-\frac{2a}{\lambda}}$
$n_1 = n_0 e^{-\frac{a}{\lambda}}$
Gate insulator
Gate electrode
$-n_{tot}$

Figure S7

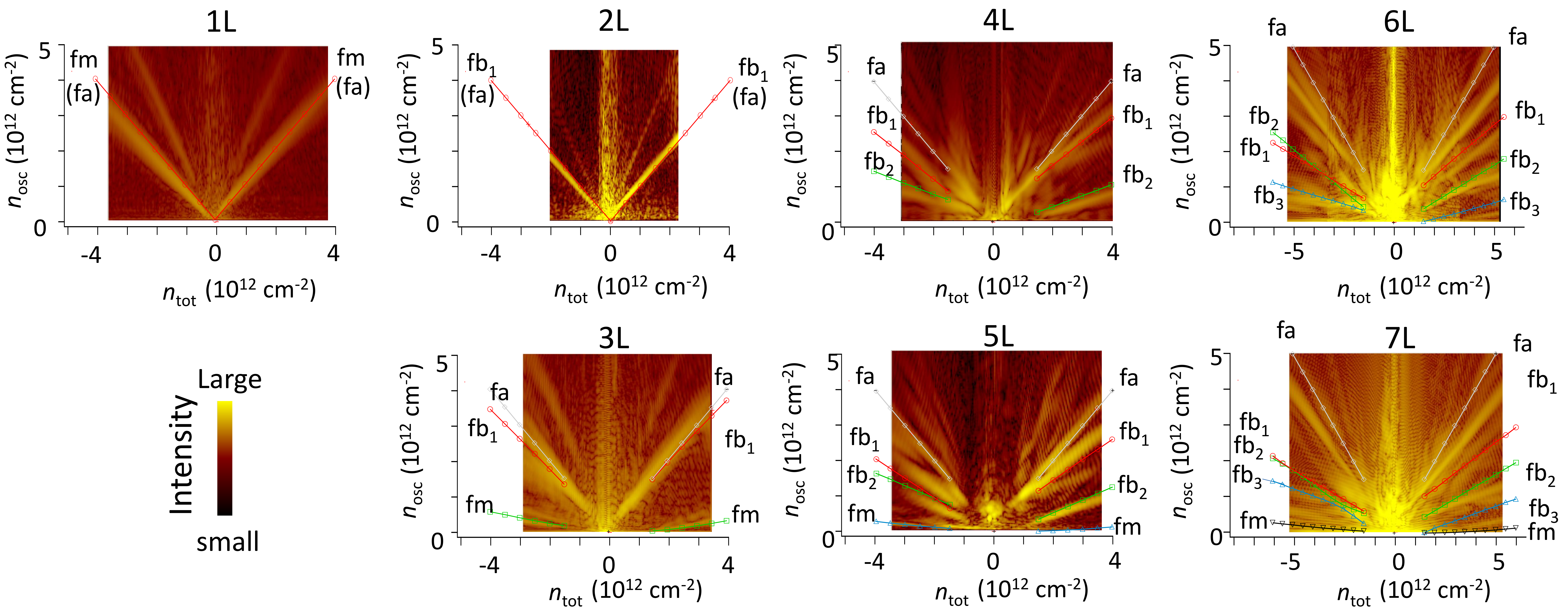

1L
2L
4L
6L
3L
5L
7L
fm
(fa)
fb1
fa
fb2
fb3
n_{osc} (10^{12} cm^{-2})
n_{tot} (10^{12} cm^{-2})
Intensity
Large
small

Fig. S8

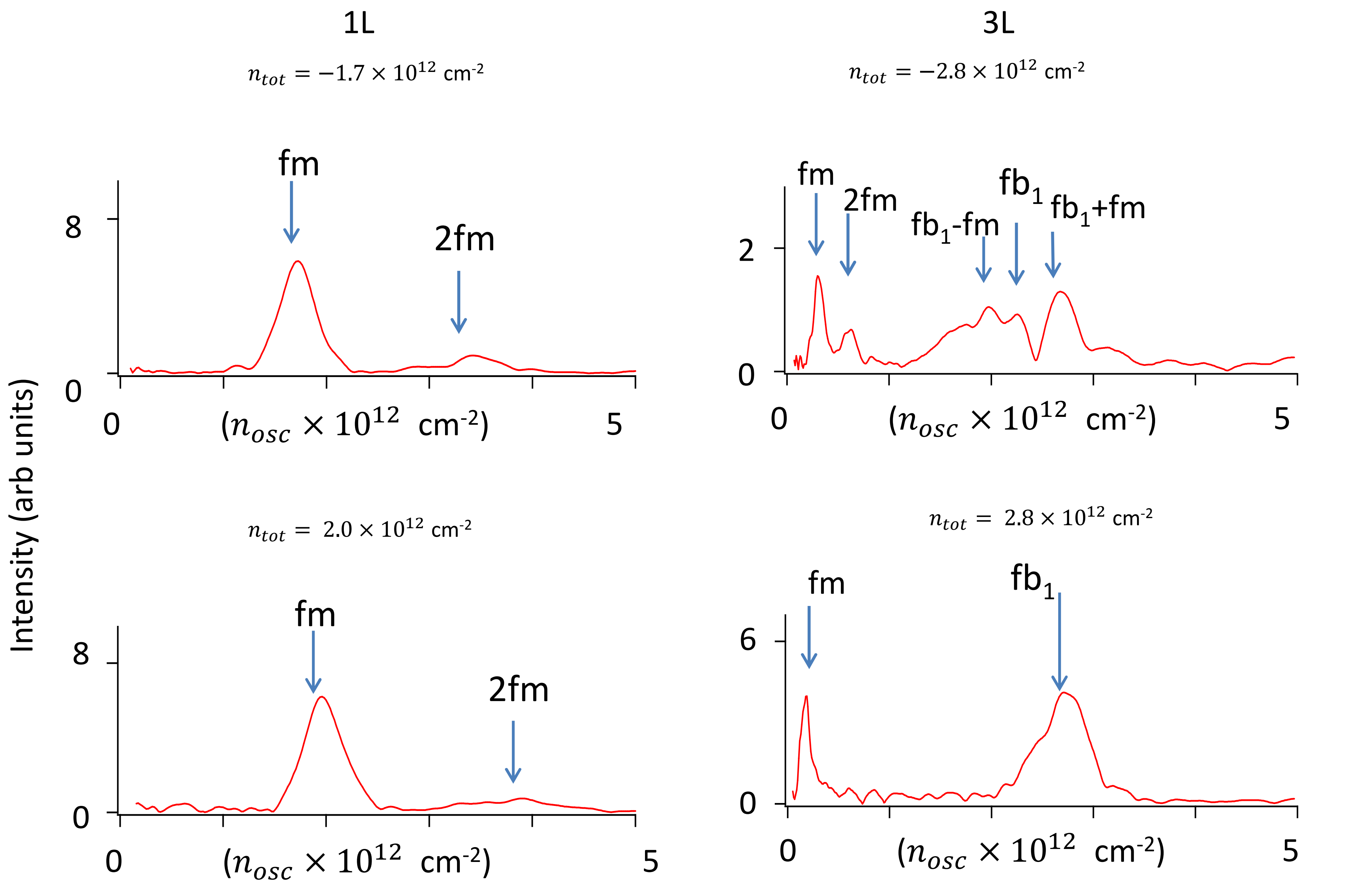

1L
3L
$n_{tot} = -1.7 \times 10^{12}$ cm^{-2}
$n_{tot} = -2.8 \times 10^{12}$ cm^{-2}
$n_{tot} = 2.0 \times 10^{12}$ cm^{-2}
$n_{tot} = 2.8 \times 10^{12}$ cm^{-2}
Intensity (arb units)
fm
2fm
fb_1-fm
fb_1
fb_1+fm
8
2
6
0
5
($n_{osc} \times 10^{12}$ cm^{-2})

Fig. S9

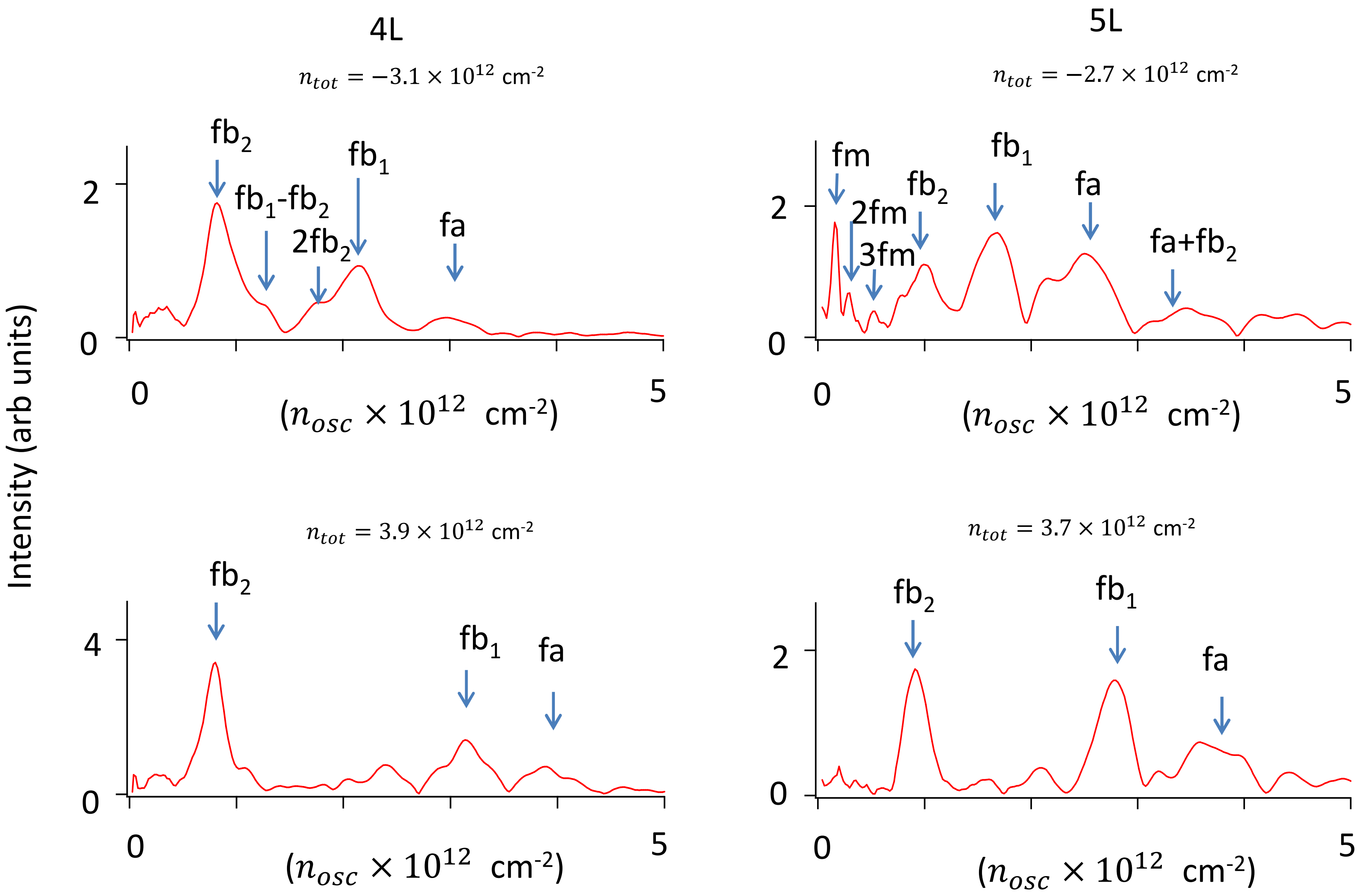

4L
$n_{tot} = -3.1 \times 10^{12}$ cm-2
fb2
fb1-fb2
2fb2
fb1
fa
5L
$n_{tot} = -2.7 \times 10^{12}$ cm-2
fm
2fm
3fm
fb2
fb1
fa
fa+fb2
Intensity (arb units)
$n_{tot} = 3.9 \times 10^{12}$ cm-2
fb2
fb1
fa
$n_{tot} = 3.7 \times 10^{12}$ cm-2
fb2
fb1
fa
($n_{osc} \times 10^{12}$ cm-2)
0
2
4
5

Fig. S10

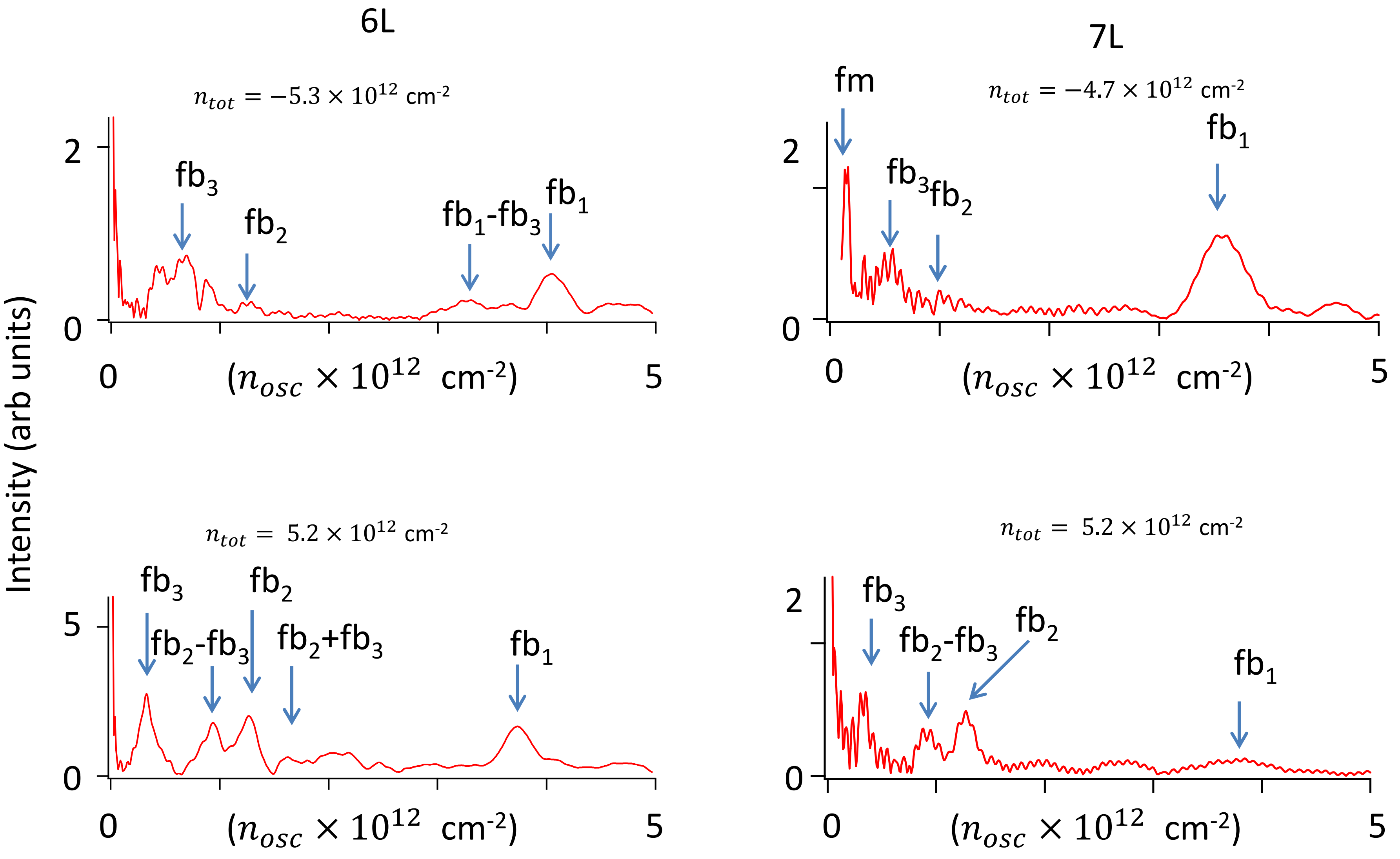

6L
7L
$n_{tot} = -5.3 \times 10^{12}$ cm^{-2}
$n_{tot} = -4.7 \times 10^{12}$ cm^{-2}
$n_{tot} = 5.2 \times 10^{12}$ cm^{-2}
$n_{tot} = 5.2 \times 10^{12}$ cm^{-2}
Intensity (arb units)
fm
fb_1
fb_2
fb_3
fb_1-fb_3
fb_2-fb_3
fb_2+fb_3
($n_{osc} \times 10^{12}$ cm^{-2})
0
2
5
